# Mechanisms of T_reg_ Suppression: Still a Long Way to Go

**DOI:** 10.3389/fimmu.2012.00191

**Published:** 2012-07-05

**Authors:** Dario A. A. Vignali

**Affiliations:** ^1^Department of Immunology, St. Jude Children's Research HospitalMemphis, TN, USA

How do natural, thymus-derived T_regs_ work? This question has preoccupied and perplexed T cell biologists for nearly two decades (Tang and Bluestone, 2008; Vignali et al., 2008; Workman et al., 2009). As with all important questions, there are some issues where there is a general consensus and others where there is still considerable disagreement. I would venture that most would agree with the following four basic tenets related to T_reg_ function.

First, T_regs_ are a critical peripheral tolerance mechanism that maintains immune homeostasis and prevents widespread autoimmunity (Ramsdell, 2003; Sakaguchi, 2003; Fontenot and Rudensky, 2005). Thus, determining how T_regs_ work is a very important goal.

Second, T_regs_ can suppress or modulate the function of a wide variety of cell populations, in diverse anatomical locations and in multiple disease situations (Tang and Bluestone, 2008).

Third, T_regs_ have an extensive arsenal and can utilize multiple contact-dependent and contact-independent mechanisms (Shevach et al., 2006; Vignali et al., 2008). This may be important because not all cell populations will be sensitive to all T_reg_ mechanisms.

Fourth, in addition to natural, thymus-derived T_regs_, there are several induced T_reg_ (iT_reg_) populations that can be generated *in vitro* or directly by T_regs_ via one of several inhibitory cytokines (TGFβ, IL-10, IL-35; Shevach, 2006; Collison and Vignali, 2008). The mechanism of suppression used by these populations seems less complicated with each appearing to depend on one key inhibitory cytokine. Thus our discussion here will focus on natural T_regs_.

However, I would argue that we know far less than we think we do about natural T_reg_ function and there is probably more contention than consensus. It is likely that continued, extensive analysis, and unique tools and approaches are likely to be required before a clear picture emerges. Some of these issues can be encapsulated around three key questions that pertain to T_reg_ function.

*Which mechanisms are most important?* This remains a contentious issue with each mechanism having its set of protagonists and antagonists. The mechanisms utilized by natural T_regs_ that have probably been examined and discussed the most are inhibitory cytokines (TGFβ, IL-10, IL-35), inhibitory receptors (CTLA4, LAG-3), cytotoxicity (Granzyme/Perforin) and metabolic disruption (IL-2 deprivation-mediated apoptosis, adenosine; Shevach et al., 2006; Tang and Bluestone, 2008; Vignali et al., 2008). Of course there may be several mechanisms that are important, with each contributing significantly in different disease scenarios, anatomical locations or against diverse cell types. Whether this is reality or appeasement remains to be resolved.

Before one determines if a mechanism is important, one would first need to obtain convincing *in vivo* evidence that a particular mechanism has a clearly definable physiologic impact. In my view, too many strong conclusions have been derived from exclusively or predominantly *in vitro* studies. Even when *in vivo* experiments have been performed, they are either limited to one model system or have potential, inherent weaknesses or caveats. It is hard to define what is “sufficient” but it seems reasonable to propose that data should be derived from multiple *in vivo* models (at least three) using (1) mice harboring conditionally (and ideally temporally) deleted alleles and (2) *in vivo* blockade/neutralization with a specific monoclonal antibody. Indeed, one could argue that no proposed T_reg_ mechanism has been extensively assessed *in vivo* thus far. Although we have championed the potential importance of IL-35 using five *in vivo* model systems with neutralizing antibodies and mutant T cell populations targeting the cytokine and its receptor (Collison et al., 2007, 2010, 2012), I would be the first to admit that more remains to be determined regarding the physiological importance of IL-35. So for mechanisms that have not reached this bar, further analysis is clearly required. Of course a major challenge is that some of these mechanisms may utilize molecules that also contribute to T_reg_ development or homeostasis. Thus it may be very difficult, or even impossible, to divorce their roles in T_reg_ function versus development/homeostasis. Furthermore, some mechanisms may be hard to target without affecting other cellular processes.

The current debate regarding the importance of one proposed T_reg_ mechanism illustrates many of these issues. T_regs_ express high levels of the high affinity IL-2 receptor (CD25; Sakaguchi et al., 1995). It was initially proposed that T_regs_ may act as a “sink” absorbing IL-2 from the local environment, thereby depriving recently stimulated T cells from the IL-2 required to initiate proliferation and subsequent differentiation. Subsequent analysis of mice lacking the capacity to make IL-2 or lacking CD25 expression revealed that IL-2 plays a critical role in maintaining peripheral T_reg_ homeostasis (D’Cruz and Klein, 2005; Fontenot et al., 2005). It was suggested that these data refuted the idea that T_regs_ act as an IL-2 “sink,” although T_reg_ function *in vivo* was not directly examined (Maloy and Powrie, 2005). More recently, it has been suggested that IL-2 deprivation-mediated apoptosis, facilitated by high CD25 expression, is a prominent mechanism of suppression used by T_regs_ (Pandiyan et al., 2007). These conclusions were supported predominantly by the observation that T cells targeted by T_regs_ die by apoptosis in *in vitro* assays but are resistant if they lack Bim, a pro-apoptotic Bcl-2 family member. Bim binds to Bcl-2 in response to stress signals, such as growth factor deprivation, thereby priming the mitochondrial pathway of apoptosis (Kuwana et al., 2005). Bim^−/−^ T cells are resistant to apoptosis induced by cytokine or growth factor withdrawal, particularly IL-2 (Bouillet et al., 1999). Curiously, Pandiyan and colleagues also showed that T_regs_ do not affect early activation or proliferation of effector T cells which is at odds with the importance of IL-2-induced STAT5 activation during these early stages (Rawlings et al., 2011).

The importance of this mechanism remains controversial as several studies have suggested that IL-2 depletion alone is not required for the suppression of human T cells (Tran et al., 2009; Vercoulen et al., 2009). More recently, we have revisited this issue to ask if T_regs_ suppress via programmed cell death pathways (Szymczak-Workman et al., 2011). Contrary to the findings of Pandiyan and colleagues, we clearly showed that T_reg_-mediated suppression of Bim^−/−^, Bim^−/−^Puma^−/−^, and Bcl-2 transgenic T cells is comparable to controls using a variety of *in vitro* and *in vivo* assays (Szymczak-Workman et al., 2011). Our use of Bim^−/−^Puma^−/−^ mice was particularly revealing as they have been shown to be completely resistant to cytokine withdrawal (You et al., 2006; Cho et al., 2009), and yet could be readily suppressed by T_regs_.

Even though *in vitro* assays can be very informative, too often strong conclusions are drawn from data obtained primarily from such assays, as highlighted above. When T cells are stimulated *in vitro*, substantial T cell apoptosis can occur if IL-2 becomes limiting, and so T cell death can be readily observed *in vitro* in the absence of T_regs_. The paradox here is that T_reg_ assays can be established where suppression is observed and either there is significant effector T cell death or almost no death, questioning whether the latter has anything to do with T_reg_ function. Although Pandiyan and colleagues suggested that Bim^−/−^ T cells were resistant to T_reg_-mediated suppression in an inflammatory bowel disease (IBD) model (Pandiyan et al., 2007), we showed in two *in vivo* models that Bim^−/−^ T cells could be effectively suppressed by T_regs_ and that Bim^−/−^ T cells had a far greater propensity to convert into iT_regs_ *in vivo* (Szymczak-Workman et al., 2011; Wang et al., 2012).

Regardless of which side of this debate your views lie, it is clear that further analysis, especially using more sophisticated *in vivo* analysis and model systems, will be required to provide further insight into this controversy. While we do not claim that T_regs_ cannot mediate suppression by any cell death pathway under any circumstances, I would respectfully argue that there is not yet sufficient data to conclude that IL-2 deprivation-mediated apoptosis is a prominent mechanism of T_reg_-mediated suppression (Pandiyan et al., 2007). Given the inextricable link between the role of CD25 in controlling T_reg_ homeostasis and its possible role in mediating suppression via IL-2 deprivation-mediated apoptosis, definitive conclusions may be hard to reach.

Given these issues, the debate over the importance of other mechanisms is likely to conjure similar discussions. Nevertheless, we need to persevere dissecting what is clearly a very important issue. A final thought on this question (sobering or exciting depending on your point of view). It remains possible that some key T_reg_ mechanisms have yet to be identified. Indeed, we have yet to define the function of many of the genes that are upregulated in T_regs_, especially when derived from disease locations (Vignali et al., 2008).

*How many mechanisms do T*_regs_ *need?* If we accept that T_regs_ may utilize many mechanisms to mediate suppression, especially at sites of substantial inflammation, it would seem important to determine how many are required to sustain credible T_reg_ function. T_reg_ depletion or absence results in the development of severe autoimmunity, but this is not matched by the disruption of any known mechanism (Fontenot and Rudensky, 2005; Vignali, 2008). This suggests that either key mechanisms have yet to be identified or multiple mechanisms work in concert to mediate T_reg_ function. I have discussed this topic in the past (Vignali, 2008), and the bottom line is that we do not know the minimum number of mechanisms required for T_reg_ function or how many can be lost before T_regs_ become fully dysfunctional.

We recently began to address this issue with surprising results. We assessed the suppressive capacity of IL-10/IL-35 double-deficient T_regs_ anticipating that they would exhibit functional defects that were greater than their single knockout counterparts. Surprisingly, IL-10/IL-35 double-deficient T_regs_ were fully functional *in vitro* and *in vivo*, essentially indistinguishable from wild type T_regs_ (Pillai et al., 2011). Thus, they seemed to have gained rather than lost function. Subsequent analysis revealed that the loss of IL-10 and IL-35 was compensated for by the concurrent increase in cathepsin E (CTSE) expression. This appeared to be required to facilitate the expression and/or release of TRAIL, a member of the TNF superfamily that can mediate apoptosis, programmed necrosis (necroptosis) or suppress proliferation via its surface bound form or as a soluble trimer (Wang and el-Deiry, 2003; Schaefer et al., 2007). Importantly, this rendered IL-10/IL-35 double-deficient T_regs_ functionally dependent on TRAIL *in vitro* and *in vivo* (Pillai et al., 2011). These data highlight two important concepts. First, the loss of certain regulatory mechanisms may result in unforeseen molecular changes which facilitate functional compensation by the upregulation of another inhibitory mechanism. Second, this study revealed that unappreciated cross-regulatory pathways may exist which control the utilization of certain suppressive mechanisms. Collectively this may serve to facilitate T_reg_ functional plasticity. Whether such mechanisms operate *in vivo* in the absence of genetic manipulation remains to be determined. However, it is possible that the mechanisms that are dominant differ in T_regs_ from different genetic backgrounds. Indeed, TRAIL is not a major mechanism used by C57BL/6 T_regs_, which express low levels of CTSE, but may be a more dominant mechanism used by Balb/c T_regs_, which coincidentally express high levels of CTSE (Pillai et al., 2011). Additional studies will clearly be required to determine the prevalence of T_reg_ functional plasticity caused by divergent genetic backgrounds and/or altered environmental circumstances.

*Is T*_reg_ *function altered in response to their environment?* While this was addressed in part in the previous section, there are two other important points to make here. First, several recent and exciting studies have highlighted the importance of specific transcription factors in shaping T_reg_ migration and function toward the control of particular Th responses (Chaudhry et al., 2009; Koch et al., 2009; Zheng et al., 2009). However, the precise molecular mechanism behind these observations remains obscure and an important priority for future research. Also, one wonders if other transcription factors play similar functions in facilitating T_reg_ control of diverse cell types and environments. Second, we have previously postulated that T_regs_ receive molecular cues from their target population and local environment that potentiates or boosts their regulatory capacity (Vignali et al., 2008). Indeed, we have shown that T_reg_:T cell target interaction can substantially boost T_reg_-mediated suppression (Collison et al., 2009; Chaturvedi et al., 2011). While the molecular mechanism of T_reg_ functional potentiation remains to be determined, it is particularly intriguing that the transcriptional landscape of “boosted” T_regs_ versus “activated” T_regs_ is very different, suggesting that a lot remains to be discovered.

In closing, while we have made substantial progress in our quest to determine how T_regs_ work, it seems that much still remains to be discovered and clarified.

## References

[B1] Bouillet P. Metcalf D. Huang D. C. Tarlinton D. M. Kay T. W. Kontgen F. Adams J. M. Strasser A. (1999). Proapoptotic Bcl-2 relative Bim required for certain apoptotic responses, leukocyte homeostasis, and to preclude autoimmunity. Science 286, 1735–1738 10.1126/science.286.5445.1735 10576740

[B2] Chaturvedi V. Collison L. W. Guy C. S. Workman C. J. Vignali D. A. (2011). Cutting edge: human regulatory T cells require IL-35 to mediate suppression and infectious tolerance. J. Immunol. 186, 6661–6666 10.4049/jimmunol.1100315 21576509 PMC3110563

[B3] Chaudhry A. Rudra D. Treuting P. Samstein R. M. Liang Y. Kas A. Rudensky A. Y. (2009). CD4+ regulatory T cells control TH17 responses in a Stat3-dependent manner. Science 326, 986–991 10.1126/science.1172702 19797626 PMC4408196

[B4] Cho Y. S. Challa S. Moquin D. Genga R. Ray T. D. Guildford M. Chan F. K. (2009). Phosphorylation-driven assembly of the RIP1-RIP3 complex regulates programmed necrosis and virus-induced inflammation. Cell 137, 1112–1123 10.1016/j.cell.2009.05.037 19524513 PMC2727676

[B5] Collison L. W. Chaturvedi V. Henderson A. L. Giacomin P. R. Guy C. Bankoti J. Finkelstein D. Forbes K. Workman C. J. Brown S. A. Rehg J. E. Jones M. L. Ni H. T. Artis D. Turk M. J. Vignali D. A. (2010). IL-35-mediated induction of a potent regulatory T cell population. Nat. Immunol. 11, 1093–1101 10.1038/ni.1952 20953201 PMC3008395

[B6] Collison L. W. Delgoffe G. M. Guy C. S. Vignali K. M. Chaturvedi V. Fairweather D. Satoskar A. R. Garcia K. C. Hunter C. A. Drake C. G. Murray P. J. Vignali D. A. (2012). The composition and signaling of the IL-35 receptor are unconventional. Nat. Immunol. 13, 290–299 10.1038/ni.2227 22306691 PMC3529151

[B7] Collison L. W. Pillai M. R. Chaturvedi V. Vignali D. A. (2009). Regulatory T cell suppression is potentiated by target T cells in a cell contact, IL-35- and IL-10-dependent manner. J. Immunol. 182, 6121–6128 10.4049/jimmunol.0803646 19414764 PMC2698997

[B8] Collison L. W. Vignali D. A. (2008). Interleukin-35: odd one out or part of the family? Immunol Rev. 226, 248–262 10.1111/j.1600-065X.2008.00704.x 19161429 PMC2631363

[B9] Collison L. W. Workman C. J. Kuo T. T. Boyd K. Wang Y. Vignali K. M. Cross R. Sehy D. Blumberg R. S. Vignali D. A. (2007). The inhibitory cytokine IL-35 contributes to regulatory T-cell function. Nature 450, 566–569 10.1038/nature06306 18033300

[B10] D'Cruz L. M. Klein L. (2005). Development and function of agonist-induced CD25+Foxp3+ regulatory T cells in the absence of interleukin 2 signaling. Nat. Immunol. 6, 1152–1159 10.1038/ni1264 16227983

[B11] Fontenot J. D. Rasmussen J. P. Gavin M. A. Rudensky A. Y. (2005). A function for interleukin 2 in Foxp3-expressing regulatory T cells. Nat. Immunol. 6, 1142–1151 10.1038/ni1179 16227984

[B12] Fontenot J. D. Rudensky A. Y. (2005). A well adapted regulatory contrivance: regulatory T cell development and the forkhead family transcription factor Foxp3. Nat. Immunol. 6, 331–337 10.1038/ni1179 15785758

[B13] Koch M. A. Tucker-Heard G. Perdue N. R. Killebrew J. R. Urdahl K. B. Campbell D. J. (2009). The transcription factor T-bet controls regulatory T cell homeostasis and function during type 1 inflammation. Nat. Immunol. 10, 595–602 10.1038/nrg2630 19412181 PMC2712126

[B14] Kuwana T. Bouchier-Hayes L. Chipuk J. E. Bonzon C. Sullivan B. A. Green D. R. Newmeyer D. D. (2005). BH3 domains of BH3-only proteins differentially regulate Bax-mediated mitochondrial membrane permeabilization both directly and indirectly. Mol. Cell 17, 525–535 10.1016/j.molcel.2005.02.003 15721256

[B15] Maloy K. J. Powrie F. (2005). Fueling regulation: IL-2 keeps CD4+ Treg cells fit. Nat. Immunol. 6, 1071–1072 10.1038/ni1105-1071 16239920

[B16] Pandiyan P. Zheng L. Ishihara S. Reed J. Lenardo M. J. (2007). CD4+CD25+Foxp3+ regulatory T cells induce cytokine deprivation-mediated apoptosis of effector CD4+ T cells. Nat. Immunol. 8, 1353–1362 10.1038/ni1536 17982458

[B17] Pillai M. R. Collison L. W. Wang X. Finkelstein D. Rehg J. E. Boyd K. Szymczak-Workman A. L. Doggett T. Griffith T. S. Ferguson T. A. Vignali D. A. (2011). The plasticity of regulatory T cell function. J. Immunol. 187, 4987–4997 10.4049/jimmunol.1102173 22013112 PMC3208127

[B18] Ramsdell F. (2003). Foxp3 and natural regulatory T cells: key to a cell lineage? Immunity 19, 165–168 10.1016/S1074-7613(03)00207-3 12932350

[B19] Rawlings J. S. Gatzka M. Thomas P. G. Ihle J. N. (2011). Chromatin condensation via the condensin II complex is required for peripheral T-cell quiescence. EMBO J. 30, 263–276 10.1038/emboj.2010.314 21169989 PMC3025460

[B20] Sakaguchi S. (2003). Regulatory T cells: mediating compromises between host and parasite. Nat. Immunol. 4, 10–11 10.1038/ni0103-10 12496970

[B21] Sakaguchi S. Sakaguchi N. Asano M. Itoh M. Toda M. (1995). Immunologic self-tolerance maintained by activated T cells expressing IL-2 receptor alpha-chains (CD25). Breakdown of a single mechanism of self-tolerance causes various autoimmune diseases. J. Immunol. 155, 1151–1164 7636184

[B22] Schaefer U. Voloshanenko O. Willen D. Walczak H. (2007). TRAIL: a multifunctional cytokine. Front. Biosci. 12, 3813–3824 10.2741/2354 17485341

[B23] Shevach E. M. (2006). From vanilla to 28 flavors: multiple varieties of T regulatory cells. Immunity 25, 195–201 10.1016/j.immuni.2006.08.003 16920638

[B24] Shevach E. M. DiPaolo R. A. Andersson J. Zhao D. M. Stephens G. L. Thornton A. M. (2006). The lifestyle of naturally occurring CD4+ CD25+ Foxp3+ regulatory T cells. Immunol. Rev. 212, 60–73 10.1111/j.0105-2896.2006.00415.x 16903906

[B25] Szymczak-Workman A. L. Delgoffe G. M. Green D. R. Vignali D. A. (2011). Cutting edge: regulatory T cells do not mediate suppression via programmed cell death pathways. J. Immunol. 187, 4416–4420 10.4049/jimmunol.1100548 21949016 PMC3197948

[B26] Tang Q. Bluestone J. A. (2008). The Foxp3+ regulatory T cell: a jack of all trades, master of regulation. Nat. Immunol. 9, 239–244 10.1038/nrg2317 18285775 PMC3075612

[B27] Tran D. Q. Glass D. D. Uzel G. Darnell D. A. Spalding C. Holland S. M. Shevach E. M. (2009). Analysis of adhesion molecules, target cells, and role of IL-2 in human FOXP3+ regulatory T cell suppressor function. J. Immunol. 182, 2929–2938 10.4049/jimmunol.0803827 19234188 PMC2777511

[B28] Vercoulen Y. Wehrens E. J. van Teijlingen N. H. de J. W. Beekman J. M. Prakken B. J. (2009). Human regulatory T cell suppressive function is independent of apoptosis induction in activated effector T cells. PLoS ONE 4, e7183 10.1371/journal.pone.0007183 19779623 PMC2746309

[B29] Vignali D. A. A. (2008). How many mechanisms do regulatory T cells need? Eur. J. Immunol. 38, 908–911 10.1002/eji.200890013 18395857

[B30] Vignali D. A. A. Collison L. W. Workman C. J. (2008). How regulatory T cells work. Nat. Rev. Immunol. 8, 523–532 10.1038/nri2343 18566595 PMC2665249

[B31] Wang S. el-Deiry W. S. (2003). TRAIL and apoptosis induction by TNF-family death receptors. Oncogene 22, 8628–8633 10.1038/sj.onc.1206125 14634624

[B32] Wang X. Szymczak-Workman A. L. Gravano D. M. Workman C. J. Green D. R. Vignali D. A. (2012). Preferential control of induced regulatory T cell homeostasis via a Bim/Bcl-2 axis. Cell Death Dis. 3, e270 10.1038/cddis.2012.9 22318539 PMC3288351

[B33] Workman C. J. Szymczak-Workman A. L. Collison L. W. Pillai M. R. Vignali D. A. (2009). The development and function of regulatory T cells. Cell. Mol. Life Sci. 66, 2603–2622 10.1007/s00018-009-0026-2 19390784 PMC2715449

[B34] You H. Pellegrini M. Tsuchihara K. Yamamoto K. Hacker G. Erlacher M. Villunger A. Mak T. W. (2006). FOXO3a-dependent regulation of Puma in response to cytokine/growth factor withdrawal. J. Exp. Med. 203, 1657–1663 10.1084/jem.20060353 16801400 PMC2118330

[B35] Zheng Y. Chaudhry A. Kas A. deRoos P. Kim J. M. Chu T. T. Corcoran L. Treuting P. Klein U. Rudensky A. Y. (2009). Regulatory T-cell suppressor program co-opts transcription factor IRF4 to control T(H)2 responses. Nature 458, 351–356 10.1038/nature07856 19182775 PMC2864791

